# Varicose Ulcers; Their Causation, Pathology and Treatment

**Published:** 1873-05

**Authors:** C. C. F. Gay

**Affiliations:** Surgeon to the Buffalo General Hospital


ART. II.—*Varicose Ulcers: their Causation, Pathology and Treatment.* By C. C. F. GAY, M. D., Surgeon to the Buffalo General Hospital.

The so-called ulcers met with in old persons, and often in the young or middle-aged, whose constitutions have become impaired from dissipation, disease, or other cause, located near the internal or external malleolus, or sometimes higher, upon the tibia, will invariably be found, I think, associated with varicose veins. The altered condition of the veins holds the relation to the ulcers of cause and effect. Indeed, whenever my attention is called to these chronic ulcers, I invariably look for enlarged veins, and always find them. The veins, when enlarged preternaturally from causes above stated, lose their valvular integrity. Whenever the calibre of veins is enlarged, the valves cease to act mechanically to maintain the column of blood contained within them, and the result is a condition of *stasis* and *pressure.* If the calibre of a vein becomes enlarged, the walls of the vein draw upon and separate the valves; or, in other words, there is no corresponding enlargement of the valve, so that several short columns of fluid become converted into one column of considerable length and greater weight; therefore that fluid, which was before normally supported by valves situated at short distances from each other, remains now measurably unsupported. The veins finally give way under the unnatural pressure, and become dilated and tortuous.

The valves of veins are said to be, by anatomists, most numerous in the veins of the lower extremities, especially of the deeper vessels, and that they are absent in the superficial veins; and hence it may be argued that valves have nothing to do toward contributing to the existence of varicose ulcers. Admitting that these smaller veins have no valves, then it may be asserted with reason that their distension comes from want of, or absence of valves, producing in either case stasis and pressure, with enfeebled circulation and breaking down of the tissue, which is dependent for its vitality and integrity upon free, rapid and unobstructed circulation. Impetus is given to venous circulation by virtue of valvular arrangement and mechanism of veins. Especially is this true during exercise. Muscular contraction over or around veins causes the blood to flow with greater velocity, provided the valves remain in their normal condition, since, while they prevent the current of blood from flowing backwards, they assist in propelling it forwards or toward the heart. Therefore the pathological condition is quite the same in either case, whether there are valves or no valves, since when valves are present they are rendered inefficient because of the preternatural dilatation of the veins,—the tissues will break down from want of proper nourishment, ultimately resulting in indolent ulceration, without any reparative power remaining to restore lost tissue. Persons whose occupations oblige them to stand long upon their feet are, it is believed, most predisposed to varicose ulcers. Persons in whom varices are present, whose occupations do not require them to stand upright much of their time, may enjoy an entire immunity, for an indefinite period of time, from the breaking away of the tissues and the formation of these troublesome lesions. That ulcers result in the case of one who has varices, while in another no ulcers appear, is explicable upon the demonstrable fact that *position* influences and increases the degree of stasis and pressure. To afford entire relief in varicocele it often becomes only necessary to support the over-loaded vessels by a suspensory bag, by prolonged use of which the vessels will return to their normal size. But palliative measures cannot be so readily and efficiently brought into requisition for the relief of over-loaded vessels and more extended columns of blood with sluggish circulation of the lower extremities; nor can the lesion under consideration, resulting therefrom, be so easily warded off and prevented; hence an operation of some kind or another, from time immemorial, has been resorted to with variable success, or, more properly speaking, perhaps, with invariable unsuccess. The best treatment for varicose veins is the best treatment for varicose ulcers. To attempt the cure of the latter without attempting the cure, or at least, mitigation of the abnormity of the former, would be a mere waste of time; hence, in approaching this lesion with intent to cure, a two-fold object must be kept in view, namely: the simultaneous cure, by the same agency, of both the varicose veins and varicose ulcers. Several methods have been in vogue and recommended by authors, three only of which I will make mention, reserving until the last allusion to that method which seems to the writer to be most worthy of trial, and which, in his hands, has never yet been attended by a single failure.

Authors heretofore have agreed in this, namely : that veins must be obliterated by some operation or other as an indispensable prerequisite to the cure of varices. I have nowhere seen the statement made that varices may be cured by the application of agencies that do not obliterate veins, but only contract them and lessen their calibre.

M. Velpeau's operation enjoys probably the best reputation for success of any operative procedure at present employed. This consists in passing a needle beneath the vein, laying a compress upon the vein and applying the twisted suture, and allowing the suture to cut its way out by ulceration.

This, or something very much like this method, is practised by Dr. Hamilton of New York, and is recommended by him in his recent and most valuable work on the "Principles and Practice of Surgery."

The most modern method, I believe, adopted for the obliteration of veins, and which is destined to meet with an ephemeral reputation, consists in the injection within the vein of the sol. ferri persulphatis. So far as I know, this is the most dangerous of all the procedures heretofore employed. Death from embolism has so often occurred from the injection of this fluid into the veins that those who were once most willing to use it have now discarded it altogether.

There are practitioners who put their entire trust and confidence in strapping. These strips of adhesive plaster must be cut from one half to three quarters of an inch in width, and of sufficient length to pass two thirds the distance around the leg, and imbricated when *in situ.* I can conceive of nothing better than these strips of adhesive plaster as a palliative means, but doubt very much their efficacy in effecting a radical cure. The application is required to be so long continued that patients could not be expected to persist in its use sufficiently long to derive any permanent benefit therefrom.

Dr. Gross gives decided preference to eschars produced by Vienna paste. He states that he is invariably successful, and that no danger attends the application. I must dissent from the view taken by this high authority as regards the danger attendant upon the application of the caustic potash. There is danger in one respect only, namely: hemorrhage. I have known hemorrhage to occur in at least two cases after the slough had been removed from the eschar, where, had it occurred in the night time or when the patient was asleep, it would have proved fatal, — the venous blood flowing in a stream like that from a vein newly opened by the lancet. The selection and preference of one of the several procedures for the radical cure of varices are influenced measurably by the success of the operating surgeon. If the employment of the ligature has been invariably attended with success and unattended with danger, there is no reason why the fortunate surgeon should seek for any method the results of which are less promising.

My own method is that recommended by Gross. Like this distinguished surgeon, I am able to say that all my cases have been successful, but must add that two of them were not altogether devoid of danger. Unless the patient be cautioned against such contingency, there may arise a venous hemorrhage that might prove disastrous. It is well, therefore, always to instruct patients how to apply the finger or make compression over the orifice, in order to arrest bleeding, in case such accident occur.

I use the potassa cum calce, rubbed up with alcohol, and apply it over the veins through an opening the size of a small pea made in adhesive plaster, taking care not to get the eschars in too close proximity, since danger might arise from two or three eschars running into each other as their surfaces enlarged, thus obtaining so large an eschar that it would be as difficult to close as the original ulcer. I usually place the eschars three or four inches apart, certainly no nearer together than this, — sometimes much further apart.

I think it unsafe to use more than five or six eschars lest erysipelas might supervene, and this number may be used simultaneously upon both legs. In twenty minutes the potash is washed off with vinegar, and the slippery-elm poultice applied over the whole leg, including eschars and ulcers. In from three to four weeks I have invariably found that the ulcers have kept pace with the eschars in the reparative process, and that both have closed ; and in not a single instance yet have I seen any return of the ulcerative process.

Of course, in treating varicose veins by this or any other method, it is not expected that all of the veins that happen to be enlarged shall be operated upon, since in many cases they are so numerous that this would be simply impossible. If a half dozen of the larger veins be operated upon, and either obliterated or partially occluded, the remaining veins return of themselves to their normal condition.

It seems to make but little difference whether the eschar is applied to the cardiac or distal side of the ulcer. Necessity, however, compels their application almost always to the cardiac side, since the ulcers are situated low upon the leg. I have met with just as good results when the ulcer was located upon the middle third of the leg by applying the eschars below the ulcer.

While writing this paper, my attention has been called by my student, Mr. Bartow, to a case of varicose ulcer upon an inmate of the alms-house, and he has watched the progress of the spontaneous cure. Ulcers, in all respects like those produced by the caustic, made their appearance directly over the most prominently dilated veins. Adhesive straps were used, the slough separated, and the reparative process now commenced, both in ulcers over the veins and the ulcer about the ancle, and continued progressively to close up until the powers of nature, unaided, save by the adhesive straps, effected a spontaneous cure.

The surgeon and physician are wise if they rightly interpret the laws of Nature, if they lend an attentive ear to her teachings, and heed her warning voice, since it is not by power or might or legerdemain that cures are wrought, but by the silent, secret, and reliable forces of nature. The case to which reference has just been made is instructive, since, so far as it goes, it points out the true method of treating varices. It will not be found unwise, I think, if the surgeon imitate this example. Ulcers treated in the manner I have indicated get well undoubtedly. I can speak with emphasis, and say that any ulcer, whatever may be its dimensions, provided only it does not encompass the leg, can be made to close up by the plan pointed out in this paper ; but now the question most naturally arises: Will the ulcers remain closed up ?

I have yet to see my first case of relapse ; nevertheless I am painfully conscious that the revolutions and revelations of time would show that the work must needs be done over again. But a few days since a gentleman presented himself at my office for advice. He had a large ulcer upon the dorsal aspect of the foot, and had also varices. He was operated upon by Dr. Hamilton twenty years ago for this same affection. The veins were tied at several points, and of course obliterated, and now, after this lapse of time and after having been subjected to the same causes which originally produced this abnormal condition, he returns for another operation, — and although the ailment has returned, there is no good reason why the operation should not be repeated, not using the needles, however, but, instead, the potassa cum calce. By its use the vessels are not obliterated, but are restored to their normal caliber, rendering the valves again efficient in their function to sustain a column of blood ; and with this changed condition the circulation is also restored in volume, velocity, and momentum.

